# Tuberculosis Treatment Monitoring by Video Directly Observed Therapy in 5 Health Districts, California, USA

**DOI:** 10.3201/eid2410.180459

**Published:** 2018-10

**Authors:** Richard S. Garfein, Lin Liu, Jazmine Cuevas-Mota, Kelly Collins, Fatima Muñoz, Donald G. Catanzaro, Kathleen Moser, Julie Higashi, Teeb Al-Samarrai, Paula Kriner, Julie Vaishampayan, Javier Cepeda, Michelle A. Bulterys, Natasha K. Martin, Phillip Rios, Fredric Raab

**Affiliations:** University of California San Diego, La Jolla, California, USA (R.S. Garfein, L. Liu, J. Cuevas-Mota, K. Collins, F. Muñoz, J. Cepeda, M.A. Bulterys, N.K. Martin, P. Rios, F. Raab);; University of Arkansas, Fayetteville, Arkansas, USA (D.G. Catanzaro); San Diego County Health and Human Services Agency, San Diego, California, USA (K. Moser);; San Francisco Department of Public Health, San Francisco, California, USA (J. Higashi);; Santa Clara County Public Health Department, San Jose, California, USA (T. Al-Samarrai);; Imperial County Public Health Department, El Centro, California, USA (P. Kriner);; San Joaquin Public Health Services, Stockton, California, USA (J. Vaishampayan)

**Keywords:** mHealth, medication adherence monitoring, smartphone, video technology, antimicrobial resistance, patient-centered care, tuberculosis and other mycobacteria, bacteria, video directly observed therapy, VDOT, California, United States

## Abstract

We assessed video directly observed therapy (VDOT) for monitoring tuberculosis treatment in 5 health districts in California, USA, to compare adherence between 174 patients using VDOT and 159 patients using in-person directly observed therapy (DOT). Multivariable linear regression analyses identified participant-reported sociodemographics, risk behaviors, and treatment experience associated with adherence. Median participant age was 44 (range 18–87) years; 61% of participants were male. Median fraction of expected doses observed (FEDO) among VDOT participants was higher (93.0% [interquartile range (IQR) 83.4%–97.1%]) than among patients receiving DOT (66.4% [IQR 55.1%–89.3%]). Most participants (96%) would recommend VDOT to others; 90% preferred VDOT over DOT. Lower FEDO was independently associated with US or Mexico birth, shorter VDOT duration, finding VDOT difficult, frequently taking medications while away from home, and having video-recording problems (p<0.05). VDOT cost 32% (range 6%–46%) less than DOT. VDOT was feasible, acceptable, and achieved high adherence at lower cost than DOT.

Tuberculosis (TB) incidence rates in the United States increased slightly in 2015 after 20 years of decline (*1*). California has the third-highest TB incidence and the most TB cases in the United States (*2*). Although TB is treatable (*3*), poor medication adherence leads to ongoing transmission, disease progression, and development of drug-resistant strains. Treating drug-resistant TB requires longer regimens with costlier, more toxic, and less effective drugs, highlighting the need for reliable treatment adherence monitoring (*4*,*5*). Strict adherence has become increasingly important because new short-course and intermittent treatment regimens have lower tolerance for adherence gaps (*6*) and because preventing acquired resistance to new drugs developed to treat multidrug-resistant (MDR) and extensively drug-resistant TB is crucial for preserving gains made in this area (*7*).

Given the severe consequences of poor adherence, health agencies recommend directly observed therapy (DOT), a process in which healthcare workers or trusted designees watch patients swallow each medication dose (*8*–*10*). Although DOT is considered the preferred method for adherence monitoring by health agencies including the World Health Organization (*11*) and the US Centers for Disease Control and Prevention (*12*), varying degrees of effectiveness have been reported from delivery of DOT through home visits by DOT workers, patients visiting clinics, and trusted family or community members performing observations (*13*). Furthermore, the DOT process itself can hinder treatment because of its high cost, personnel requirements, potential for stigma, impact on patient income and mobility, and travel required by patients or healthcare workers (*14*).

These barriers to DOT prompted some US TB programs to use videoconferencing technology through videophones, computers, or smartphones to remotely observe patients swallowing pills (*15*,*16*). This live (synchronous) approach became known as video directly observed therapy (VDOT). Studies of synchronous VDOT indicate that patients adhere to their regimens and mostly prefer VDOT over in-person DOT and that VDOT saves TB programs money by reducing travel and personnel costs (*17*–*19*). However, barriers such as limiting observation to business hours, network interruptions, and requirements of the Health Insurance Portability and Accountability Act (HIPAA) prompted development of smartphone applications to enable recorded (asynchronous) VDOT. A pilot study in Kenya provided the first published evidence of asynchronous VDOT’s acceptance (*20*). Subsequently, the first study to systematically evaluate asynchronous VDOT among TB patients in San Diego, California, and Tijuana, Mexico, showed that patients and providers found VDOT to be feasible and acceptable, with >95% of expected doses observed, but lacked a comparison group (*21*). We assessed treatment adherence for patients using VDOT versus traditional DOT and evaluated adherence, feasibility, acceptability, and cost differences between urban and rural TB programs.

## Methods

### Design

We conducted a prospective, multisite, single-arm trial in which all participants had TB treatment monitored using asynchronous VDOT. As a comparator, medical record reviews provided adherence data from a sample of patients who were monitored using in-person DOT at the same clinics. All VDOT participants used DOT for the first 2 weeks or until medication tolerance was established (whichever was longer) before initiating VDOT. Participants continued using VDOT until treatment completion or their provider switched them back to DOT.

A University of California–San Diego Institutional Review Board approved this study, as did each participating health department. Study participation did not affect treatment prescribed by participants’ physicians.

### Population and Recruitment

The study population consisted of patients receiving DOT for active or suspected pulmonary TB in 3 urban (San Diego, San Francisco, Santa Clara) and 2 rural (San Joaquin, Imperial) California health jurisdictions. Patients >18 years of age with no plans to move from the jurisdiction before completing treatment and >30 days of treatment remaining were eligible. Patients with MDR TB were eligible; however, only 1.4% of California’s TB patients had MDR TB (*22*).

TB program staff recruited patients sequentially during routine DOT visits. Research staff explained VDOT and study procedures to interested patients and obtained written informed consent. Asynchronous VDOT was available only to study participants; patients who declined participation continued treatment through DOT. In San Diego and Santa Clara counties, synchronous VDOT was also offered to patients who were unsuitable for DOT. Two patients declined to participate before enrollment, and 5 who initially consented withdrew before starting VDOT.

Historical controls (n = 159) were group-matched by age, race or ethnicity, and sex from a random sample of patients at the 5 study sites to obtain estimates of adherence to in-person DOT. To avoid selection bias from using patients who were not offered VDOT, controls were selected from patients who completed TB treatment during the year before asynchronous VDOT introduction at each site.

### VDOT Description

**Figure 1:**
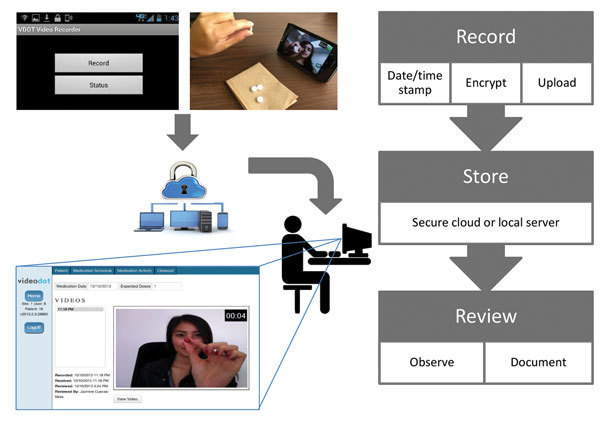
Schematic of asynchronous VDOT in a study assessing VDOT for monitoring tuberculosis treatment, 5 California health districts, 2015–2016. Patients use VDOT smartphone application to record a video of themselves ingesting their medications. After recording stops, the application encrypts the video and transfers it through a cellular or Wi-Fi connection to a server for storage and playback. On a routine basis, treatment monitors log into a secure website to view each video and document their observations. Missing videos or videos not showing complete dose ingestion trigger follow-up procedures to investigate missed doses and provide patient support as needed. VDOT, video directly observed therapy.

The VDOT application (Figure 1) enabled participants to record themselves swallowing each treatment dose and send videos for review by a DOT worker. Each recorded dose was automatically date- and time-stamped, encrypted, and uploaded to a secure server over a cellular or wireless network. Once the data were received by the server, the smartphone application deleted videos from the device to prevent unintentional disclosure of participant information and conserve device memory. Videos were stored on the smartphone in a manner that prevented viewing, editing, resending, or deleting them to protect participant privacy and ensure video fidelity. The asynchronous design allowed participants to take their medications regardless of network connectivity (e.g., while traveling) because videos uploaded automatically whenever cellular or WiFi connections were established. An application status screen allowed participants to see when videos were uploaded or pending. The system sent daily medication reminders by text message or email. Participants were loaned smartphones with cellular data plans to ensure that the application performed identically for all participants and avoided service outages.

TB program staff trained participants to use VDOT during routine clinic or home visits. As with DOT, whenever possible, participants were seen by staff who spoke their preferred language; otherwise, telephone-based translation services were used. Once participants demonstrated VDOT competency, they were given smartphones and instructed to record their next dose alone at the prescribed time. If the participant or DOT worker had concerns about the procedures, the DOT worker kept the phone and repeated the training during subsequent in-person DOT visits; thus, the number of training days could vary by participant. Participants also received a VDOT reference pamphlet. To minimize health risks, participants were instructed to call or visit their healthcare provider before taking medications when side effects occurred, rather than reporting side effects through videos. DOT workers regularly logged onto a password-protected website to view videos and document their observations. If expected videos were missing or videos did not clearly show participants ingesting medications, participants were contacted to identify problems and provide support as needed. Decisions about returning participants to DOT were made on a case-by-case basis rather than by using strict adherence-based criteria because some missed doses were unavoidable and requiring DOT for participants who could not meet in-person might adversely affect adherence. Routine medication refill and health monitoring visits occurred per standards of care.

### Data Collection

We conducted brief (15–20 minute) baseline (before initiating VDOT) and follow-up (after ending VDOT) telephone interviews to assess sociodemographic variables, experience using mobile technology, TB history and risk factors, privacy concerns, and perceptions of TB treatment monitoring. Research staff, rather than care providers, conducted interviews to minimize response bias. Participants received $10 USD for each interview; no remuneration was paid for sending videos.

To measure treatment adherence among control patients, TB program staff reviewed their DOT records abstracting treatment start and end dates; DOT start and end dates; treatment outcome; and the number of doses expected, observed, and unobserved (i.e., self-administered, not taken, or treatment suspended). DOT was predominantly community-based and required staff travel; however, San Francisco also offered clinic-based DOT to patients who could conveniently access the clinic. Nonclinical personnel conducted most DOT visits; nurses also provided some DOT based on clinical needs and staffing considerations. Control patients were not interviewed.

### VDOT versus DOT

Because VDOT was introduced after participants had initiated treatment using DOT, we calculated the fraction of expected doses observed (FEDO) while the patient was on VDOT as a measure of adherence. FEDO equals the number of observed doses divided by the sum of observed doses, missed doses, and self-administered doses. For each day that medication doses were expected, DOT workers documented whether they observed all, some, or no pills being ingested. For this analysis, doses were only considered observed if all pills were taken. If no video was received or ingestion of fewer than all pills was observed, the dose was considered missed, as were self-administered doses. Because weekend doses are not ordinarily observed in DOT, they were excluded from this calculation. FEDO was calculated for VDOT and control patients. Adherence (doses observed divided by total doses prescribed) was also computed for control patients because they used DOT throughout their treatment.

### Cost Analysis

Employing a healthcare provider perspective, we used an ingredients-based, bottom-up approach (*23*,*24*) to estimate the average per-patient cost of a standard, Centers for Disease Control and Prevention–recommended (*3*), 6-month treatment regimen for drug-susceptible TB using DOT or VDOT. Because the likelihood of medication side effects differs between the intensive (56 daily doses) and continuation (126 daily doses) phases of treatment, costs were stratified by treatment phase and then summed to calculate the overall average patient treatment cost. Nurses from 4 sites completed a standardized questionnaire assessing personnel time, personnel salaries, and resources required to administer DOT and VDOT. Staff turnover precluded data collection from the fifth site. Completed questionnaires were discussed jointly by teleconference to ensure that all sites interpreted the questions and responded uniformly. Cost data were collected during March–June 2017 and presented in 2017 USD.

DOT personnel costs included time for patient contact, administrative tasks, and travel. VDOT personnel costs included time for community-based visits before initiating VDOT, patient VDOT training, administrative tasks, video observation, and follow-up when expected videos were not received. Some in-person observations also occurred among patients using VDOT because all patients received DOT for >2 weeks before starting VDOT and patients in San Francisco were observed in-person during weekly medication refill visits to the clinic. We converted annual salaries, including fringe benefits, into an hourly rate, assuming a 40-hour workweek. All personnel reported full-time employment. The total time for each DOT-related task (administrative, patient contact, and travel) needed to treat each patient was multiplied by the hourly rate and then summed for all personnel.

To calculate an overall per-patient travel cost, we multiplied the average number of miles per patient visit and total number of in-person visits by the current federal mileage reimbursement rate ($0.54 per mile). This approach conservatively estimated travel costs because it assumed that DOT workers used personal vehicles rather than costlier county-owned vehicles. Because DOT workers often visited multiple patients in a single outing rather than returning to the health department between each patient visit, we calculated the average number of miles per visit by dividing the average number of miles driven per day for DOT-related activities by the average number of in-person visits on any given day.

Corporate prices paid by the investigators for smartphones ($100) and service plans ($54/month) during the study were applied to all sites. An estimated VDOT application cost of $35/month/patient was applied on the basis of products commercially available at the time this article was written. Costs of antibiotics, laboratory tests, chest radiographs, and clinical examinations were excluded because they were assumed to be equivalent for VDOT and DOT.

### Statistical Analysis

We used Kruskal-Wallis and Fisher exact tests to determine differences in sociodemographic characteristics, TB history, TB risk factors, and VDOT perception variables across study sites. We assessed associations between FEDO and independent variables by using Kruskal-Wallis tests (categorical variables), Wilcoxon rank sum tests (binary variables), and Spearman correlation coefficients (continuous variables). We used simple linear regression to identify factors associated with FEDO and considered significant variables (p<0.15) for inclusion in multivariable linear regression analysis. We used backward stepwise elimination to remove nonsignificant variables until only variables with p<0.05 remained in the final model and assessed normal assumption of residuals by using normal probability plot, and influential observations were assessed by residuals and Cook’s distance. We performed Wilcoxon rank sum tests to compare FEDO between VDOT and DOT and used R statistical software (*25*) to conduct analyses.

## Results

### Participant Characteristics and VDOT Perceptions

Overall, 274 participants (248 urban and 26 rural) enrolled during October 2014–October 2015 contributed adherence and baseline interview data (Table 1). Median participant age was 44 (range 18–87) years; 61% were male, 57% were Asian, 30% were Hispanic or Latino, and 7% were white. Most (67%) were born in other countries (predominantly countries in Asia), followed by the United States (17%) and Mexico (16%). Education and income were low overall, but most participants had health insurance. Most participants (90%) owned cell phones; 72% owned smartphones. Substance use, other than smoking (42%), was uncommon, and no participants were homeless. Only race or ethnicity, education level, and country of birth differed across sites (p<0.05).

**Table 1:** Baseline characteristics of patients participating in a study assessing VDOT for monitoring tuberculosis treatment, by site, 5 California health districts, 2015–2016*

Characteristic	Total	Site					p value†
		San Diego	San Francisco	Santa Clara	Imperial	San Joaquin	
No. patients	272	99	99	49	10	15	
Age, y							
Mean (SD)	43.8 (16.5)	42.0 (16.9)	46.5 (15.5)	42.2 (16.2)	46.6 (22.0)	41.7 (16.1)	0.19
Range	18–87	18–87	24–86	21–83	21–69	21–76	
Education							
<Primary school	26 (10)	10 (10)	12 (12)	3 (6)	1 (10)	0	0.02
High school	105 (39)	39 (40)	39 (40)	13 (27)	5 (50)	9 (60)	
Some college or technical school	67 (25)	25 (26)	17 (18)	15 (31)	4 (40)	6 (40)	
>Bachelor’s degree	71 (26)	24 (24)	29 (30)	18 (37)	0	0	
Sex							
M	167 (61)	59 (60)	61 (62)	34 (69)	5 (50)	8 (53)	0.65
F	105 (39)	40 (40)	38 (38)	15 (31)	5 (50)	7 (47)	
Race or ethnicity							
Asian	154 (57)	41 (41)	68 (69)	37 (76)	10 (10)	7 (47)	<0.001
Caucasian or white	19 (7)	7 (7)	7 (7)	1 (2)	0	4 (27)	
Hispanic or Latino	82 (30)	42 (42)	21 (21)	6 (12)	9 (90)	4 (27)	
Other‡	17 (6)	9 (9)	3 (3)	5 (10)	0	0	
Country of birth							
United States	47 (17)	22 (22)	9 (9)	4 (8)	4 (40)	8 (53)	<0.001
Mexico	44 (16)	26 (26)	7 (7)	5 (10)	5 (50)	1 (7)	
Other§	181 (67)	51 (52)	83 (84)	40 (82)	1 (10)	6 (40)	
Annual household income, $USD							
<10,000	110 (44)	43 (46)	43 (47)	13 (29)	6 (55)	8 (57)	0.09
10,000–30,000	74 (30)	24 (28)	29 (32)	15 (33)	3 (27)	3 (21)	
30,000–50,000	26 (10)	13 (15)	9 (10)	2 (4)	0 (0)	2 (14)	
>50,000	39 (16)	10 (11)	11 (12)	15 (33)	2 (18)	1 (7)	
Had health insurance, yes vs. no	229 (85)	76 (78)	85 (86)	43 (90)	10 (91)	15 (100)	0.12
Owned cell phone, yes vs. no	247 (90)	90 (91)	92 (93)	44 (90)	9 (82)	12 (80)	0.33
Owned smartphone, yes vs. no	196 (72)	71 (72)	67 (68)	41 (84)	7 (64)	10 (67)	0.26
Homeless, yes vs. no¶	0	0	0	0	0	0	NA
Ever smoked cigarettes, yes vs. no	116 (42)	43 (43)	41 (41)	17 (35)	7 (64)	8 (53)	0.41
Marijuana use, yes vs. no¶	18 (7)	5 (5)	7 (7)	2 (4)	2 (18)	2 (13)	0.26
Noninjection drug use, yes vs. no¶	4 (1)	1 (1)	2 (2)	1 (2)	0	0	1
Ever injection drug use, yes vs. no	3 (1)	1 (1)	0	1 (2)	1 (9)	0	0.07

*Values are no. (%) participants unless otherwise indicated. VDOT, video directly observed therapy; NA, not applicable. †p values based on Fisher exact test or Kruskal-Wallis test. Variable totals might not sum to column totals because of missing data. ‡Other race group includes African American (n = 3), American Indian (n = 2), Pacific Islander (n = 1), and mixed and other races (n = 11). §Other countries were predominantly in Asia. ¶Referent period is the previous 6 months.

We obtained VDOT observation data from the 274 enrolled participants, 214 (78%) of whom completed follow-up interviews (Table 2). Twenty-seven percent of participants reported not sharing their VDOT experience with family members, and 66% did not share with others. Although 34% disclosed having concerns about being seen recording VDOT videos, only 8% failed to record >1 dose because of privacy concerns. At follow-up, only 2% of participants thought VDOT was less confidential than DOT, and 96% reported that VDOT was “very or somewhat easy to perform”; only 3% would choose DOT over VDOT if they had to repeat treatment, and 96% would recommend VDOT to other patients. Training VDOT procedures to participants took a median of 1 day across sites; 74% of participants required 1 day, whereas 4% needed >4 days (data not shown). Only 12 (4.4%) participants were returned to DOT before completing treatment because of poor adherence (n = 5), a lost or broken phone (n = 4), or technical or connectivity problems (n = 3).

**Table 2:** Reported experiences of patients participating in a study assessing VDOT for monitoring tuberculosis treatment, by site, 5 California health districts, 2015–2016*

Characteristic	Total	Site					p value†
		San Diego	San Francisco	Santa Clara	Imperial	San Joaquin	
No. patients	274‡	100	99	49	11	15	
VDOT use							
Months on VDOT, median (IQR)	5.4 (3.5–7.1)	5.2 (3.2–6.3)	5.4 (3.5–7.3)	5.5 (4.1–8.1)	4.0 (2.1–5.6)	6.1 (4.4–7.7)	0.08
FEDO, median (SD), IQR	93.0 (13.5), 83–97	88.7 (15.1), 77–94	95.5 (11.8), 87–98	95.2 (10.3), 89–98	84.5 (20.0), 78–94	96.1 (7.9), 93–98	<0.001
No. patients in follow-up interviews	214	74	84	39	9	7	
Tuberculosis and treatment perceptions							
Did you share your VDOT experience with family members?							
Yes	156 (73)	55 (74)	55 (66)	30 (77)	9 (100)	6 (86)	0.18
No	58 (27)	19 (26)	29 (34)	9 (23)	0	1 (14)	
Did you share your VDOT experience with friends, neighbors, classmates, or coworkers?							
Yes	73 (34)	24 (32)	28 (33)	13 (33)	3 (33)	5 (71)	0.38
No	141 (66)	50 (68)	57 (67)	26 (67)	6 (67)	2 (29)	
Were you concerned someone would see you using the VDOT cell phone?							
Yes	73 (34)	19 (26)	34 (40)	15 (38)	4 (44)	1 (14)	0.23
No	141 (66)	55 (74)	51 (60)	24 (62)	5 (56)	6 (86)	
Did you ever fail to record a video because you were worried someone was watching you?							
Yes	18 (8)	7 (9)	9 (11)	2 (5)	0	0	0.87
No	196 (92)	67 (91)	76 (89)	37 (95)	9 (100)	7 (100)	
Confidentiality of VDOT vs. DOT?							
More	146 (70)	49 (67)	55 (66)	30 (77)	7 (78)	5 (83)	0.68
Less	5 (2)	2 (3)	1 (1)	2 (5)	0	0	
Same	59 (28)	22 (30)	27 (33)	7 (18)	2 (22)	1 (17)	
VDOT experience							
Overall, how easy/difficult did you find the VDOT process?							
Very easy	174 (81)	58 (78)	68 (79)	36 (92)	6 (67)	6 (86)	0.19
Somewhat easy	32 (15)	14 (19)	13 (15)	3 (8)	1 (11)	1 (14)	
Somewhat or very difficult	9 (4)	2 (3)	5 (6)	0	2 (22)	0	
If you had to redo tuberculosis treatment, would you choose VDOT or DOT?							
VDOT	192 (90)	67 (92)	75 (87)	35 (90)	9 (100)	6 (86)	
DOT	6 (3)	1 (1)	4 (5)	1 (3)	0	0	0.9
No preference	16 (7)	5 (7)	7 (8)	3 (8)	0	1 (14)	
Would you recommend VDOT to other tuberculosis patients?							
Yes	202 (96)	70 (95)	81 (96)	35 (97)	9 (100)	7 (100)	0.95
No	8 (4)	4 (5)	3 (4)	1 (3)	0	0	
How often did you take tuberculosis medication away from home?							
Never or rarely	120 (56)	39 (53)	54 (64)	19 (49)	5 (56)	3 (43)	0.36
Less than half or half the time	48 (22)	18 (24)	12 (14)	13 (33)	2 (22)	3 (43)	
Most of the time or every time	46 (21)	17 (23)	19 (22)	7 (18)	2 (22)	1 (14)	
How often did you have problems using the VDOT application?							
Never	82 (38)	24 (32)	41 (48)	16 (41)	1 (11)	0	0.06
Rarely	99 (46)	35 (47)	33 (39)	20 (51)	5 (56)	6 (86)	
Less than half the time	23 (11)	9 (12)	9 (11)	2 (5)	2 (22)	1 (14)	
Half the time or more	10 (5)	6 (8)	2 (2)	1 (3)	1 (11)	0	
How often did poor reception cause you problems uploading videos?							
Never	65 (31)	13 (18)	34 (40)	12 (31)	3 (33)	3 (43)	
Rarely	103 (49)	41 (56)	35 (42)	20 (51)	3 (33)	4 (57)	0.15
Less than half the time	24 (11)	11 (15)	7 (8)	3 (8)	3 (33)	0	
Half the time or more	20 (9)	8 (11)	8 (10)	4 (10)	0	0	

*Values are no. (%) participants unless otherwise indicated. DOT, directly observed therapy; FEDO, fraction of expected doses observed = number of complete doses observed via VDOT divided by the number of doses expected; IQR, interquartile range; VDOT, video directly observed therapy. †p values based on Fisher exact test or Kruskal-Wallis test. Variable totals might not sum to column totals because of missing data. ‡Includes 2 participants who used VDOT but had missing baseline interview data.

### FEDO by Treatment Monitoring Method

**Figure 2:**
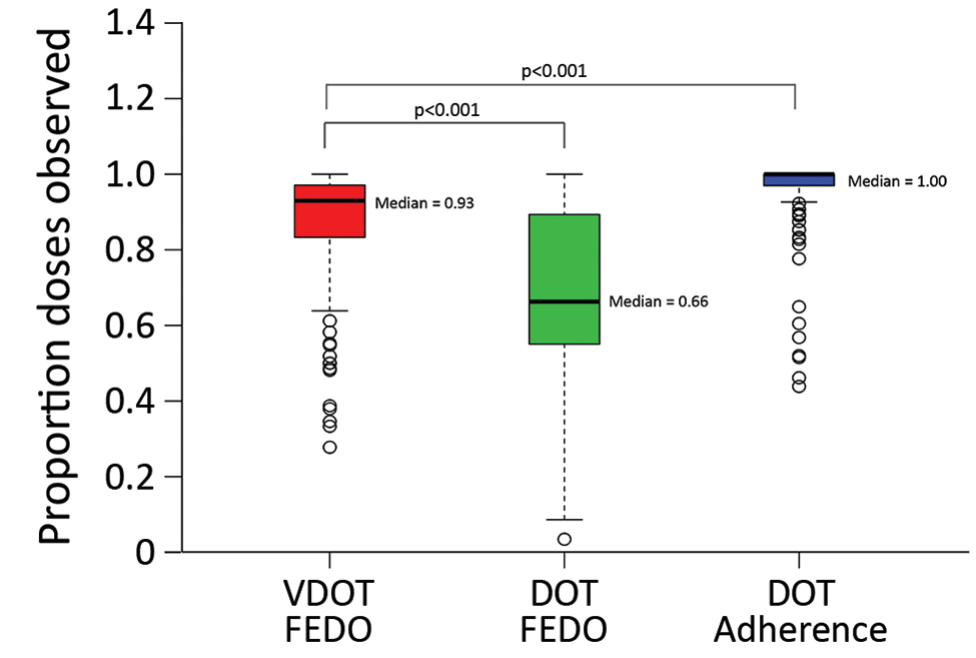
FEDO among patients monitored ingesting medication for tuberculosis by VDOT compared with FEDO and adherence for patients monitored using in-person DOT in a study assessing VDOT for monitoring tuberculosis treatment, 5 California health districts, 2015–2016. FEDO assessed by number of complete doses observed through VDOT divided by the number of doses expected. Adherence assessed by number of doses observed through DOT divided by the number of prescribed doses. Because missed or self-administered doses had to be rescheduled, the number of times a dose was expected could exceed the number of doses prescribed. DOT, directly observed therapy; FEDO, fraction of expected doses observed; VDOT, video directly observed therapy.

Study participants used VDOT a median of 5.4 months (interquartile range [IQR] 3.5–7.1 months), generating 42,211 videos (Table 2). Median FEDO was 93.0% (IQR 83.4%–97.1%), compared with 66.4% (IQR 55.1%–89.3%) for control patients using only DOT (Figure 2). By contrast, median adherence was 100% (IQR 97.0%–100%) for control patients because of an unwavering commitment by TB program staff to ensure patients completed their treatment.

### Correlates of FEDO

Median FEDO differed across individual sites (range 84.5%–96.1%; p<0.001); however, the extreme values occurred in the 2 rural sites (Table 2). Thus, FEDO did not differ between the combined urban and rural sites (92.8% vs. 94.2%; p = 0.51) in bivariate analysis (Table 3). FEDO differed by race or ethnicity and country of birth, increased with longer VDOT use and higher annual income, and decreased with marijuana use in the prior 6 months. Participants who found VDOT more difficult, more often took medications while away from home, more often had problems using the VDOT application, and more often had problems uploading videos because of poor network connectivity had lower FEDOs.

**Table 3:** Bivariate analysis of FEDO by participant characteristics and perceptions of VDOT in a study assessing VDOT for monitoring tuberculosis treatment, by selected characteristics, 5 California health districts, 2015–2016*

Characteristic	No. patients	FEDO, median (SD)	p value†
Demographic and socioeconomic			
Site type			
Urban county	248	92.8 (13.3)	0.51
Rural county	26	94.2 (15.4)	
Age, y, correlation coefficient	272	−0.03	0.63
Months on treatment, correlation coefficient	272	−0.14	0.02
Education			
<Primary school	26	88.4 (13.7)	0.17
High school	105	93.0 (14.5)	
Some college or technical school	67	93.9 (13.2)	
>Bachelor’s degree	71	94.2 (11.9)	
Sex			
M	167	92.8 (12.7)	0.70
F	105	93.4 (14.8)	
Race or ethnicity			
Asian	154	95.0 (10.8)	<0.001
Caucasian or white	19	95.6 (15.4)	
Hispanic or Latino	82	86.8 (16.3)	
Other‡	17	87.0 (11.4)	
Country of birth			
United States	47	90.3 (17.2)	<0.001
Mexico	44	84.7 (16.0)	
Other§	181	94.5 (10.7)	
Annual household income, $ (USD)			
<10,000	110	92.7 (13.6)	0.04
10,000–30,000	74	90.8 (14.8)	
30,000–50,000	26	92.0 (10.4)	
>50,000	39	96.3 (9.8)	
Insured at baseline			
Yes	229	93.4 (13.6)	0.28
No	42	89.9 (13.4)	
Owned cell phone at baseline			
Yes	247	93.0 (12.9)	0.49
No	26	93.1 (18.3)	
Owned smartphone at baseline			
Yes	196	92.8 (13.2)	0.84
No	76	93.6 (14.5)	
Tuberculosis risk factors			
Ever smoked cigarettes			
Yes	116	92.3 (12.3)	0.26
No	157	93.6 (14.4)	
Marijuana use in previous 6 mo			
Yes	18	88.1 (19.3)	0.01
No	252	93.2 (13.0)	
Tuberculosis and treatment perceptions			
Did you share your VDOT experience with family members?			
Yes	156	92.9 (14.1)	0.80
No	58	93.1 (13.0)	
Did you share your VDOT experience with friends, neighbors, classmates, or coworkers?			
Yes	73	93.4 (13.7)	0.48
No	141	92.7 (13.9)	
Were you concerned someone would see you using the VDOT cell phone?			
Yes	73	93.3 (14.9)	0.89
No	141	92.6 (13.2)	
Did you ever fail to record a video because you were worried someone was watching you?			
Yes	18	89.2 (10.3)	0.28
No	196	93.1 (14.1)	
Confidentiality of VDOT vs. DOT?			
More	146	92.9 (14.4)	0.92
Less	5	92.7 (12.5)	
Same	59	93.0 (12.7)	
VDOT experience			
Overall, how easy/difficult did you find the VDOT process?			
Very easy	174	93.6 (12.4)	0.001
Somewhat easy	32	91.7 (14.6)	
Somewhat or very difficult	9	66.9 (19.8)	
If you had to redo tuberculosis treatment, would you choose VDOT or in-person DOT?			
VDOT	192	93.2 (13.9)	
In-person DOT	6	91.3 (13.3)	0.55
No preference	16	92.1 (12.9)	
Would you recommend VDOT to other tuberculosis patients?			
Yes	202	92.7 (13.9)	0.42
No	8	94.8 (9.8)	
How often did you take tuberculosis while medication away from home?			
Never or rarely	120	94.2 (12.3)	0.04
Less than half or half the time	48	93.4 (13.8)	
Most of the time or every time	46	88.9 (16.4)	
How often did you have problems using the VDOT application?			
Never	82	95.1 (13.7)	0.001
Rarely	99	92.8 (10.3)	
Less than half the time	23	88.2 (12.3)	
Half the time or more	10	74.3 (25.7)	
How often did poor reception cause you problems uploading videos?			
Never	65	95.8 (15.6)	0.01
Rarely	103	92.4 (11.5)	
Less than half the time	24	89.6 (8.7)	
Half the time or more	20	84.9 (19.3)	

*DOT, directly observed therapy; FEDO, fraction of expected doses observed = number of complete doses observed through VDOT divided by the number of doses expected; VDOT, video directly observed therapy. †p values based on Wilcoxon rank sum test, Kruskal-Wallis test, or Spearmen correlation coefficient. ‡Other race groups were African American (n = 3), American Indian (n = 2), Pacific Islander (n = 1), and mixed and other races (n = 11). §Other countries were predominately in Asia.

In multivariable analysis (Table 4), higher FEDO was independently associated with longer duration of VDOT use. Lower FEDO was associated with birth in Mexico or the United States compared with other countries; feeling VDOT was somewhat or very difficult compared with very easy; taking medication away from home most or every time compared with never; and having problems using VDOT more than half the time compared with “never.”

**Table 4:** Multivariable linear regression analysis of factors associated with FEDO among patients treated for tuberculosis, 5 California health districts, 2015–2016*

Characteristic	Beta coefficient	SE	p value
Months on VDOT (per month)	0.008	0.003	0.01
Country of birth (referent: other)			
Mexico	−0.095	0.022	<0.001
United States	−0.048	0.022	0.03
Perceived ease or difficulty of VDOT (referent: very easy)			
Somewhat easy	−0.003	0.024	0.90
Somewhat or very difficult	−0.130	0.042	0.002
Took medications while away from home (referent: never or rarely)			
Less than half or half the time	−0.004	0.020	0.83
Most of the time or always	−0.049	0.021	0.02
Had problems using the VDOT application (referent: never)			
Rarely	−0.001	0.018	0.97
Less than half the time	−0.040	0.029	0.16
More than half the time	−0.220	0.041	<0.001


*FEDO, fraction of expected doses observed = number of complete doses observed through VDOT divided by the number of doses expected; VDOT, video directly observed therapy.

### VDOT versus DOT Costs

The estimated cost for monitoring a 6-month treatment regimen using VDOT (Table 5) varied by site (range $3,031–$3,911) and was 6%–46% cheaper than community-based DOT (range $3,212–$5,788) across sites. Reduced personnel costs drove savings, which offset smartphone-related costs.

**Table 5:** Average in-person DOT and VDOT costs per treatment course, by site, based on standard drug-susceptible tuberculosis treatment regimen consisting of 56 intensive-phase and 126 continuation-phase doses, 4 California health districts, 2015–2016*

Characteristic	San Diego	San Francisco	San Joaquin	Imperial
In-person DOT costs				
Personnel				
Administrative tasks	1,038	3,043	1,913	842
In-person patient contact	1,207	622	2,223	656
Travel	1,939	1,065	702	1,293
Total personnel (% of total)	4,185 (91)	4,729 (97)	4,838 (84)	2,791 (87)
Mileage (% of total)	364 (9)	158 (3)	950 (16)	421 (13)
Grand total	4,549	4,888	5,788	3,212
VDOT costs				
Personnel				
Administrative tasks	796	1,922	1,771	1,183
In-person patient contact	671	291	393	346
Watching videos	80	131	152	473
Other, e.g., training and follow-up	869	933	156	348
Total personnel (% of total)	2,526 (79)	3,277 (88)	2,472 (83)	2,350 (78)
Mileage (% of total)	20 (1)	0	31 (1)	46 (2)
Smartphone costs, device and service (% of total)	424 (13)	424 (7)	424 (8)	424 (14)
VDOT application service fee, $35/mo/patient (% of total)	210 (7)	210 (5)	210 (7)	210 (7)
Grand total	3,179	3,911	3,137	3,031
% Change for VDOT versus in-person DOT				
Personnel costs, %	−40	−31	−49	−16
Overall costs, %	−30	−20	−46	−6


*All values are USD unless otherwise indicated. Comparable data could not be obtained from the Santa Clara site because of staff turnover. DOT, directly observed therapy; VDOT, video directly observed therapy.

## Discussion

VDOT was feasible and acceptable for monitoring TB medication ingestion in urban and rural California health districts. A higher proportion of expected doses was observed as scheduled among VDOT participants than among in-person DOT participants, resulting in shorter treatment duration.

Median FEDO for VDOT was lower than previously reported (95%) (*21*), possibly because the earlier study oversampled low-risk patients during the first trial of the VDOT application. Alternatively, the disparity could be attributable to our conservative approach to calculating FEDO by only counting doses when all pills were taken and treating all doses as missed when a software error caused 5% of videos received to be unviewable. If unviewable videos and partial doses were counted as observed, FEDO in our study (median 96.4%, IQR 89%–99%) would have matched the prior study.

Defining eligibility criteria for VDOT a priori appears unnecessary. Despite efforts to define VDOT eligibility criteria using these data, only 1 variable known before treatment (country of birth) was associated with FEDO. DOT studies similarly found few predictors of adherence (*26*). Initial concerns about older patients or those unfamiliar with smartphones having difficulty using VDOT were unfounded because these factors did not predict FEDO. Travel was also not problematic; study staff often reported that patients who traveled or had nontraditional work hours could adhere better after switching to VDOT. Monitoring anti-TB therapy involves ongoing communication, negotiation, and cooperation between patients and healthcare providers (*27*,*28*), and patient-centered care increased when patients had VDOT as an option. Other than ensuring that patients could tolerate their medications, operate the VDOT application, and access smartphones and service, no evidence was found to support requiring other eligibility criteria.

We observed an association between VDOT problems and lower FEDO, which was driven by only 10 participants who reported having problems half the time or more. Similarly, the association between FEDO and difficulty using VDOT resulted from 9 participants reporting that VDOT was somewhat or very difficult. However, most of these participants encountered the software error described previously, which lowered their FEDO and could explain why they felt VDOT was difficult. Lower FEDO among participants taking medications away from home most or every time could be attributable to difficulty finding a private location to make videos while away from home, which might have also made DOT difficult.

Unlike DOT or synchronous VDOT, asynchronous VDOT enabled patients to take medications outside normal business hours (e.g., at mealtimes or bedtime), which could minimize side effects and improve adherence (*16*). VDOT also allowed participants to fast during religious holidays, because medication doses could be observed at night after fasting ended. Avoiding intermittent dosing by allowing observations after hours and on weekends and holidays through VDOT could also improve treatment efficacy (*29*). All sites, except 1, included participants with MDR TB (VDOT duration range 30–537 days) whose adherence was comparable to the cohort overall. Because MDR tuberculosis patients at times require dosing more than once daily, VDOT reduced stress on the TB programs and facilitated quicker return to daily activities for patients on these much longer regimens. Additionally, asynchronous VDOT does not require consistent network connectivity, making it useful for patients in remote areas.

Although asynchronous VDOT offers greater flexibility and reduces self-administered doses, DOT and synchronous VDOT might allow more frequent patient–provider interaction and facilitate patient support. However, asynchronous VDOT could improve case management efficiency by shifting the focus of in-person visits from treatment monitoring, perceived by patients as punitive (*30*,*31*), to patient care, support, and other key TB program activities such as contact tracing. The appropriate mix of remote monitoring and direct interaction to support patients throughout treatment remains to be determined with further research. Cost-effectiveness studies are also needed to inform policies around treatment monitoring.

TB risk factors were self-reported and could be underestimated if participants chose not to disclose stigmatized behaviors. Because no patients were homeless, we could not examine this risk factor. Three sites (Santa Clara, Imperial and San Joaquin) had never used asynchronous VDOT previously, potentially promoting conservative patient selection; however, their results were similar to sites with VDOT experience. In addition, San Francisco differed from the other sites by requiring weekly, rather than monthly, refill visits, which could have increased adherence; however, adherence was comparable across sites. Because providers could switch participants from VDOT back to DOT, observed FEDOs could have been skewed upward if nonadherent participants were removed from VDOT early. However, only 12 (4.3%) participants returned to DOT before completing treatment, of whom only 5 did so because of poor adherence. Removing these participants had little effect on FEDO overall. This study was conducted in a high-income country and might not reflect VDOT performance in low- and middle-income countries.

To our knowledge, our study is the largest prospective study of asynchronous VDOT to date. Patients with TB treatment monitored by VDOT had more expected medication doses observed than patients monitored using DOT. VDOT performed similarly in urban and rural health departments, with high observation rates and positive patient perceptions across sites. Although some participants returned to DOT, most were effectively monitored to completion by using VDOT. VDOT reduced TB-control program costs compared with DOT. Other than country of birth, patient characteristics did not predict adherence, suggesting that TB-control programs could offer VDOT broadly and provide additional support, or switch to DOT if adherence declines rather than restricting VDOT use to patients with prespecified characteristics. Asynchronous VDOT was found to be a cost-effective method of monitoring TB treatment in the United States; however, similar studies are needed in countries with high burdens of TB and limited resources, where smartphone penetration and cultural acceptance of transmitting personal images over the Internet could differ.
